# Exploring dietitians’ practice and perspectives on the role of dietary patterns during cancer treatment: A qualitative study

**DOI:** 10.1371/journal.pone.0302107

**Published:** 2024-05-14

**Authors:** Annie R. Curtis, Nicole Kiss, Katherine M. Livingstone, Robin M. Daly, Anna Ugalde

**Affiliations:** 1 Institute for Physical Activity and Nutrition, Deakin University, Geelong, VIC, Australia; 2 Allied Health Research, Peter MacCallum Cancer Centre, Melbourne, VIC, Australia; 3 School of Nursing and Midwifery, Deakin University, Geelong, VIC, Australia; University of Glasgow MRC/CSO Social and Public Health Sciences Unit, UNITED KINGDOM

## Abstract

**Background:**

Dietitians are nutrition professionals equipped with specialised skills required to prevent and treat malnutrition in cancer. Optimisation of dietary intake is recommended as the primary nutrition strategy for the treatment of cancer-related malnutrition. However, it is unclear whether dietary patterns, described as the combination, quantity, and frequency of food consumption, are considered. This study examined dietitians’ current food-based management of malnutrition; explored dietitians’ awareness of dietary patterns and assessed barriers and enablers to the use of dietary patterns in clinical practice.

**Methods:**

This qualitative study consisted of semi-structured interviews with oncology dietitians. Dietitians were recruited through national nutrition societies, social media, and professional networks. Audio-recorded interviews were transcribed verbatim and analysed using inductive thematic analysis.

**Results:**

Fourteen oncology dietitians from across four Australian states and territories participated. Three themes were identified: (i) principles to guide nutritional care, (ii) dietary patterns as a gap in knowledge and practice, and (iii) opportunities for better care with systems as both a barrier and enabler. Dietetic practice was food-focussed, encouraging energy and protein-rich foods consistent with nutrient-focussed evidence-based guidelines. Dietitians encouraged one of two nutrition-related approaches, either encouraging intake of ‘any tolerated food’ or ‘foods supportive on longer-term health’. Dietitians were generally unaware of dietary patterns and questioned their relevance in certain clinical situations. A multidisciplinary team approach, adequate food service and dissemination of dietary patterns research and education were identified as opportunities for better patient care.

**Conclusions:**

Recommendations for the treatment of malnutrition vary between oncology dietitians and uncertainty exists regarding dietary patterns and their relevance in clinical practice. Further exploration into the role of dietary patterns to treat cancer-related malnutrition and education for dietitians are required prior to implementation of a dietary patterns approach into clinical practice.

## Introduction

Optimising nutritional status is essential to prevent adverse cancer-related outcomes. Malnutrition and muscle loss are common complications of cancer and are associated with reduced or early termination of treatment, reduced time to remission, poorer quality of life and decreased survival [1–5]. Malnutrition and muscle loss affect up to 60% of people with cancer due to tumour and treatment-related side-effects that impact nutritional intake and metabolism [6]. The treatment of cancer-related malnutrition and muscle loss aims to optimise nutritional intake and participation in physical activity [7]. Therefore, international evidence-based guidelines recommend all people who are identified as ‘at risk’ of malnutrition via nutritional screening, or have a cancer diagnosis or treatment protocol placing them at high nutritional risk, should be referred to a dietitian for a comprehensive nutritional assessment and provision of appropriate nutritional treatment [8,9].

Individualised nutrition therapy has previously been shown to improve nutritional intake, physical function, quality of life and survival in hospitalised patients with cancer [10]. Personalised dietary counselling is the first line treatment for cancer-related malnutrition, with the aim to increase oral intake and meet heightened energy and nutrient requirements [9]. This approach is commonly termed a ‘food-first’ approach. A ‘food-first’ approach is defined as the optimization of dietary intake through increased consumption or fortification of foods and beverages, before advancing to other medical nutrition strategies such as oral nutrition supplements, enteral nutrition (i.e., tube feeding) or parenteral nutrition (i.e., intravenous nutrition).

In line with a ‘food-first’ approach, dietary patterns describe the complexity of human dietary exposure, encompassing the combination, quantity and frequency of food consumption, the nutrient content of those foods, and the interaction between dietary components [11]. Dietary patterns rich in energy and protein may have a beneficial role in reducing the odds of malnutrition after cancer diagnosis [12,13]. For example, a dietary pattern characterised by high oily fish and nut intake was associated with reduced odds of malnutrition in 2,415 adults previously diagnosed with cancer [13]. However, this evidence is only emerging and further research is required to identify an optimal dietary pattern for people with cancer. Dietary pattern assessment methods are broadly categorised into index-based (*a priori*) or data-driven (*a posteriori*) methods. *a priori* methods assess adherence to any pre-defined diet quality indices and commonly align with national dietary guidelines [14,15]. *A posteriori* methods use statistical techniques to derive new dietary patterns from dietary data at hand [14–16].

After diagnosis people with cancer are often motivated to make dietary changes [17]. However, dietary changes are often small and not necessarily clinically relevant, suggesting a need for expert nutritional support [18]. In high-income countries it is estimated that between 30 to 50% of people with cancer and cancer survivors receive dietetic care, a service that is strongly desired by this group of individuals [19,20]. Previous research has demonstrated that dietitians who provide care to people with head and neck cancer demonstrate high rates of awareness and implementation of evidence-based nutrition guidelines [21]. However, it remains unclear how dietitians treat malnutrition in practice.

A ‘food-first’ approach is recommended to treat malnutrition; however, it remains unclear how dietitians implement this dietary approach in the clinical setting. With emerging evidence supporting a positive role for specific dietary patterns to support nutritional status and muscle health in cancer [13,22], it is essential to understand dietitians’ current practice and perception of dietary patterns to successfully integrate dietary patterns approaches into clinical practice. This study aimed to understand dietetic management of malnutrition and low muscle mass in the clinical oncology setting, focussing on food-specific recommendations, and to examine dietitians’ awareness and understanding of dietary patterns and barriers and enablers to their use in clinical practice.

## Methods

### Study design

This is a qualitative study which used semi-structured interviews to collect detailed information pertaining to the study aims. This study was approved by the Deakin University Human Research Ethics Committee (HEAG-H 6_2022) on 31^st^ March 2022. Prior to providing consent, all participants were provided with a plain language statement which outlined the study purpose and roles and responsibilities of participants, as well as the researchers’ contact information. Informed consent was obtained from all participants prior to commencing each interview, either in writing (provision of an electronic signature confirming participation) or verbally (voice recorded verbal consent to participate) during interview recordings. Research procedures and reporting were conducted in accordance with the Consolidated Criteria for Reporting Qualitative Research guidelines (S1 Table) [23]. Participants were not provided with a copy of their transcript for comment or correction. Each participant was provided with a de-identified plain language summary of the study findings. One author (ARC) had access to participant’s identifiable information during and after data collection for the purpose of participation organisation and dissemination of the plain language summary of study findings.

### Participants and recruitment

Recruitment occurred between June 2022 and January 2023. Eligible participants were qualified dietitians who had a minimum of one years’ experience in the nutritional care of adults with cancer, and who currently worked in an Australian public or private healthcare setting; for example, public or private hospitals and private clinical practices. In the first instance, recruitment occurred via dissemination of study information through national nutrition societies (Nutrition Society of Australia [NSA] and Australian Society for Parenteral and Enteral Nutrition [AuSPEN]) and via researchers’ social media platforms (i.e., Twitter). In addition, a researcher’s (NK) professional networks were used to identify suitable dietitians and these dietitians were contacted via email by one researcher (ARC). Sampling was purposive with a focus on dietitians from various Australian states and territories and levels of professional experience, this approach was used to gain a broad understanding of the research aims from a heterogeneous sample of oncology dietitians.

### Data collection

Interviews were conducted using video conferencing technology (Zoom) by one researcher (female, Accredited Practising Dietitian and PhD candidate) who was trained in qualitative research methods (ARC). Interviews were organised via email communication with participants and conducted at a time which best suited them. The researcher (ARC) and participants were the only people present during each interview. In most cases, the researcher and participant did not have an existing relationship. All participants were instructed that they could decline at any time for any reason before providing consent. Data were collected using a purpose-designed, semi-structured interview guide (Table 1), including a brief sociodemographic questionnaire (Table 2). Response prompts and follow up questions were used to explore emerging themes or gather detailed information on the topics of interest. The interview guide was edited by all authors, including dietitians with experience in the clinical oncology setting, and informed by the study aims. Each interview lasted for approximately 30 minutes. All interviews were audio recorded and transcribed verbatim using artificial intelligence software (Otter.ai Los Altos, CA). Interview transcripts were manually read and corrected by one researcher (ARC). Brief field notes were collected during the interviews; however, these were not subsequently used during data analysis.

**Table 1:** Semi-structured interview questions and prompts.

1	What is your current role? Describe where you work, your role and the patient group you work with.
2	Can you describe your nutritional management of malnutrition, low muscle mass and sarcopenia? • Prompt: Describe the nutrient goals for this patient group. • Prompt: Which nutritional guidelines do you refer to?
3	Can you describe the specific foods or types of foods you recommend to patients when managing malnutrition, low muscle mass and/or sarcopenia? • Prompt: specific diet or dietary patterns?
4	Can you describe your understanding of what dietary patterns are?
5	Based on your experience can you describe whether dietary patterns are used in the nutritional management of malnutrition, low muscle mass and/or sarcopenia in the clinical setting where you work? • Prompt: if used, what foods characterise this dietary pattern? • Prompt, if not used, why is that the case?
6	What is your understanding of the role of dietary patterns for the management of malnutrition, low muscle mass and/or sarcopenia? • Prompt: What evidence do we currently have to support this? • Prompt: Why do you think it is important?
7	Can you describe any barriers you foresee to implementing a holistic dietary patterns approach to managing malnutrition, low muscle mass and/or sarcopenia in the clinical setting where you work? • Prompt: what would prevent you from using a dietary patterns approach
8	Can you describe any enablers you foresee for implementing a holistic dietary patterns approach to managing malnutrition, low muscle mass and/or sarcopenia in the clinical setting where you work? • Prompt: what would support you to use a dietary patterns approach
9	How receptive to implementing a new approach to nutritional management (such as a holistic dietary pattern approach) do you consider your current workplace to be?
10	What would need to occur to implement a holistic dietary patterns approach?

**Table 2:** Brief demographics questionnaire.

1	Are you an accredited practising dietitian (APD)?
2	Which Australian state or territory do you currently practise dietetics?
3	Do you work in a public or private healthcare setting?
4	Do you work in a metropolitan, rural, or regional health care setting?
5	How many years have you been practising in an oncology setting?
6	What proportion of your clinical workload is oncology?
7	Which tumour stream do you most commonly work in?

### Data analysis

This was a qualitative study and transcribed interview data was used to conduct inductive thematic analysis. Thematic analysis was used to guide the identification and development of key themes; this was conducted using a six phase process outlined previously [24]. The first three interview transcripts were read in their entirety by three authors (ARC, NK and AU) who collaboratively conducted manual line-by-line inductive open coding of the text. The remaining interview transcripts were read and subjected to line-by-line inductive open coding by one author (ARC). Identified codes were then presented and discussed with two additional authors (NK and AU), where excerpts from the text were examined. Discussions took place until consensus regarding context and meaning could be reached, and all authors accepted that the findings were representative of the data. Salient concepts began to emerge during this process, at which point codes were grouped into initial themes. Themes were discussed between three authors (ARC, NK and AU), defined and named accordingly. Data analysis and data collection were conducted concurrently, and recruitment was ceased once data saturation had been achieved. The expected sample size was also informed by our previous qualitative research including health professionals interviews [25–27] and recommendations from the literature [28]. Qualitative description was used to examine and interpret participant’s understanding of dietary patterns and current dietetic practice. De-identified quotes have been provided to support this process. Reflexive practices occurred throughout the data collection and analysis period, with the interviewer (ARC), an Accredited Practicing Dietitian and PhD candidate, verbally reflecting on her subjectivity with other authors. As a fellow dietitian, the interviewer (ARC) was cognisant of the biases that this may introduce to the data collection or analysis processes. As such, reflective discussions about participants practices and perspectives were had between the interviewer (ARC) and other authors (NK and AU). Descriptive statistics were used to summarise participant demographic characteristics. NVIVO software was used to manage the data and Microsoft Excel was used to present themes (and categories), codes and quotes before manuscript preparation.

## Results

### Participants

Fourteen dietitians participated in the interviews from four Australian states and territories. Seventeen dietitians initially responded to an invitation to participate; however, three dietitians were not contactable to schedule an interview. Twelve dietitians represented metropolitan (86%) and two represented regional (14%) healthcare services. Eleven dietitians worked in a public healthcare setting (79%), two in private (14%) and one in both public and private settings (7%). Dietitians primarily provided care for patients with head and neck (n = 4; 29%), gynaecological (n = 2; 14%), haematological or gastrointestinal cancers (n = 1; 7%), or a combination of cancer types (n = 6; 43%). Experience as an oncology dietitian ranged from four to 20 years (median: 6 years). The median duration of interviews was 28 minutes (range: 18–41 minutes).

Three key themes were identified: (i) principles to guide nutritional care, (ii) dietary patterns as a gap in knowledge and practice; and (iii) opportunities for better care with systems as both a barrier and enabler. Themes and corresponding categories are presented with supporting quotes. Quotes are assigned in the text using participant identification numbers (PT 1 to PT 14).

#### Theme 1: Principles to guide nutritional care

Theme 1 reflected agreeance between evidence-based guidelines for malnutrition and dietitians’ recommendations in clinical practice. Diversity in regard to approaches used by dietitians to address malnutrition was analysed under this theme. Two categories supported this theme: (i) concordance with practice and recommendations; and (ii) diversity in approaches to nutritional care.

*Concordance with practice and recommendations*. All dietitians reported providing nutritional recommendations consistent with evidence-based nutrition guidelines. Dietitians referenced guidelines such as ‘Evidence based practice guidelines for the nutritional management of cancer cachexia’ [29] and ‘European Society for Clinical Nutrition and Metabolism (ESPEN) guidelines on nutrition in cancer patients’ [9] to support their clinical decision making. Increasing energy and protein intake was consistently recommended to manage patients with–or at risk of–malnutrition:

“*If I notice that a patient’s intake is inadequate*, *I give that education on high energy high protein*. *That’s my stock standard with literally every patient I see*.*”* (PT 14)

In practice, dietitians referred to using a ‘food-first’ approach to nutritional care. Dietitians stated that they preferred to enhance energy and protein intake using foods and beverages, before escalating to other strategies such as oral nutrition supplements and enteral nutrition strategies:

“*In terms of intervention*, *we always start off with a food-first approach*, *looking at higher calorie*, *high protein food options available within the hospital and implementing or recommending those*.*”* (PT 6)

Dietitians emphasised that their patients also preferred a ‘food-first’ approach, rather than relying on intake of oral nutrition supplements to support their nutritional intake:

“*We do prefer to do food strategies first*. *Okay*, *maybe some extra snacks*, *fortify meals*. *Yeah*, *a lot of the patients like to use real foods*, *real whole foods*, *real natural foods*, *rather than going for those commercial supplements or any other supplements*. *So*, *we tend to do that first*. *For some patients who are really*, *you know*, *already sort of past that point*. *It’s supplements*.*”* (PT 8)

Dietitians consistently spoke about the need to tailor their recommendations to the patient’s unique clinical situation and dietary preferences. In consultation with the patient, dietitians reported making dietary recommendations that built on current intake:

“*It’d be looking at what the patient’s original diet is initially and then developing ideas with the patients that are building on their existing diet to increase energy and/or protein value of the diet*.*”* (PT 11)

Others noted that in the oncology setting it is about improving rather than perfecting diet:

“*…you’re not often reaching that magical 100% of estimated requirements*, *but it’s about working within the context of that patient’s symptoms and side effects and things that we can adjust to help get a little bit more energy or protein in*.*”* (PT 3)

*Diversity in approaches to nutritional care*. The perceived optimal approach to achieving adequate nutritional intake was dichotomous, being divided into two major approaches. Some dietitians prioritised the intake of any tolerated foods and beverages, acknowledging common treatment-related side effects such as poor appetite as barriers to dietary intake. Referring to the time period during active treatment, one dietitian stated:

“*Any calorie is a good calorie at this time*.*”* (PT 14)

Dietitians who were inclined to follow this approach to achieving adequate dietary intake prioritised their patient’s food preferences:

“*I tend to be at the school of thought of if it works for that patient at that time*, *so I’m not very judgmental on healthy fats or non-healthy fats*, *healthy foods versus non-healthy foods*, *I just tend to run with if the patient tolerates ice cream*, *and that’s what they can do at that point in time*.*”* (PT 12)

It was noted of the acute treatment period when risk of malnutrition is greatest:

“*it’s only a short time just to get you through chemo or just to get you through radiotherapy*, *we can focus on the healthy eating post treatment*. *Because really*, *you do just want to minimise that loss of weight and just get some energy into them*.*”* (PT 8)

The alternative approach prioritised the intake of foods which may be beneficial to longer term health:

“*A lot of [patients] have heard that you have got to keep your weight on and ‘should I be having ice cream and all really high sugar*, *high calorie type stuff’*. *But for me*, *I try to switch it around to explaining where else you can get good quality energy from*, *the balance of those meals*, *and sources of protein explaining where that comes from*.*”* (PT 10)

Another dietitian noted:

“*To be honest with you*, *I’m not overly keen on our high energy*, *high protein approach anyway*. *Like some of the things that’s in our handouts*, *I’m like*, *you know*, *they’re not nutritious foods*. *And I know it’s only short term*, *but at the same time*, *it’s like*, *should we really be promoting it*?*”* (PT 8)

Dietitians reported prescribing a wide range of foods and beverages for patients in order to achieve adequate energy and protein intake. For example, one dietitian stated:

“*If it is an animal or it comes from one*, *then that’s what we try and focus on there*.*”* (PT 7)

Other energy and protein-rich foods that were commonly recommended by dietitians included nuts and nut butters, dairy foods (milk and yoghurts), olive oil, avocado, tofu, beans, lentils, chickpeas, and discretionary foods (ice-cream and sweets). Multiple dietitians reported that their food-based recommendations were primarily supported by general knowledge of energy and protein-rich foods. In explanation of why certain foods are recommended in their practice, one dietitian stated:

“*…generally*, *it’s probably coming from probably long-standing knowledge from uni days and hasn’t been updated since then*.*”* (PT 12)

Theme 1 demonstrated whilst dietitians referred to nutrient-focussed evidence-based guidelines to guide their practice, the food-based strategies used by dietitians varied significantly.

#### Theme 2: Dietary patterns as a gap in knowledge and practice

Theme 2 reflected a varied understanding of dietary patterns and how they could be used in relevant clinical situations. Two categories supported this theme: (i) Ambiguity regarding dietary patterns and their alignment with current dietary recommendations; and (ii) Dietary patterns may not always be relevant in practice.

*Ambiguity regarding dietary patterns and their alignment with current dietary recommendations*. Unlike nutrient-focussed recommendations which prescribe optimal nutrient targets for people with cancer, dietary patterns consider the foods and beverages, and their respective nutrient contents, to achieve these targets. In line with nutrient-focused evidence-based guidelines [9], dietitians’ specific food-based recommendations were primarily guided by the nutrient composition (i.e., high energy and protein) of the foods. The majority of dietitians articulated that their focus was to enhance energy and protein intake. Regarding the focus of their current dietary recommendations to manage malnutrition, one dietitian stated:

“*It’s probably more the energy and protein rather than specific food groups as such…”* (PT 11)

Dietitians expressed a very general understanding of dietary patterns which varied widely between participants. One dietitian summarised dietary pattens as a way to define a patient’s whole dietary intake:

“*So dietary patterns*, *describing just the whole*, *the entirety of a person’s eating*. *So rather than saying that they’re having a high fat or a high carbohydrate*, *low carbohydrate diet*, *you’re sort of more describing the totality of their diet*.*”* (PT 5)

Other dietitians associated dietary patterns with the variety of food consumed in the diet:

“*I probably think about [dietary patterns] as that variety in the diet*. *And probably*, *I guess*, *my main thought*, *I always go back to the Australian Guide to Healthy Eating food groups*. *So having that variety*, *having the regularity of meals and snacks across the day*.*”* (PT 11)

Some dietitians broadly reflected on well-known a-priori dietary patterns such as the Mediterranean diet. Regarding the use of a dietary pattern approach in practice, one dietitian stated:

“*I definitely talk about high energy high protein diets*, *but we never sort of talk about Mediterranean or anything of that sort of style*.*”* (PT 1)

Other dietitians felt they were already recommending dietary patterns in practice, However, were instead referring to meal patterns including the preferred timing and frequency of meals and snacks:

“*Probably from the pattern point of view*, *it’s pretty common that small regular meals are the recommended way to go*…*”* (PT 7)

*Dietary patterns may not always be relevant in practice*. Dietitians were concerned that recommending a dietary pattern may not be appropriate in certain clinical situations. For example, some dietitians perceived dietary patterns as being not prescriptive enough for some patients:

“*I think talking about more dietary patterns is great*. *I think sort of talking more about ways of eating is better than being too prescriptive*. *But at the same time*, *I also think that the barrier is some patients are at a point where they’re like*, *just tell me what to do*. *And it is sometimes easier to go you need to eat one tub of yoghurt*, *one glass of milk and have one extra piece of banana bread per day on top of what you’re doing*. *And it’s like*, *okay*, *I can cope with that*. *I think if I’m talking sort of too big a picture for some patients*, *some patients are not at a point in their treatment where they’re ready to be thinking bigger picture*. *It’s a treatment phase not a lifestyle phase of the cancer journey*.*”* (PT 1)

Another dietitian commented on the need to prioritise any foods that the patient can consume when treatment-related side effects are prevalent:

“*To be honest*, *in the population that I look after*, *I often don’t refer to [dietary patterns]*. *Because purely I think the barriers to nutrition intake are so significant that it’s often about whatever we can get into their mouth rather than choosing a specific pattern*.*”* (PT 2)

Alternatively, other dietitians spoke positively about the use of dietary patterns in the cancer survivorship period to improve cancer-related outcomes such as cancer survival and reducing cancer recurrence.

“*I think there’s huge work probably in the survivorship space around dietary pattern for those patients post treatment or in the survivorship stage*. *To help reduce their risk of recurrence…other chronic diseases*. *I think there’s definitely a huge role for dietary patterns in that space*.*”* (PT 6)

Overall, dietitians expressed limited understanding of dietary patterns with varied perceptions of the definition of a dietary pattern and how it may or may not be relevant to practice, especially during the acute treatment period.

#### Theme 3: Opportunities for better care with systems as both a barrier and enabler

Theme three captured the barriers and enablers to providing optimal nutrition care within various healthcare settings, including considerations for implementing a dietary patterns approach into practice. Two categories supported this theme: (i) Multidisciplinary approaches to optimal nutritional management; and (ii) Healthcare systems provide barriers and enablers to nutritional management.

*Multidisciplinary approaches to optimal nutritional management*. Dietitians expressed the benefits of working within a multidisciplinary team to appropriately manage malnutrition. A number of limitations to achieving optimal nutrition management were reported by dietitians. Some dietitians highlighted concerns regarding appropriate and timely referrals in their healthcare setting:

“*Unfortunately*, *quite a few patients are missed due to issues with screening and referral practices that I’m having trouble addressing*.*”* (PT 5)

Other dietitians expressed the need for all members of the multidisciplinary team to be unified in their communication around nutrition:

“*I think that in the first few weeks after a big surgery there’s a general fear and reluctance to have a lot of fibre containing foods*. *Fruits and vegetables*. *Maybe with good reason*. *Maybe a little excessive sometimes the concern expressed by surgeons*.*”* (PT 5)

Similarly, dietitians reported that other members of the multidisciplinary team held strong nutrition-related beliefs which were at times not supported by adequate evidence and were incongruent with other dietary recommendations:

“*There have been some doctors who have sort of strong dietary beliefs*. *So*, *vegan for everyone as one example*. *I guess it’s that buy in from everyone in the team relaying that same message…”* (PT 7)

In one of the represented healthcare settings, a dietitian noted that the nutritional management of patients was isolated due to issues with access and clinician engagement:

“*It’s very dietitian-only unfortunately*, *it’s not really a great multidisciplinary team approach*. *Like we’ve got no access to exercise [physiology]*, *we don’t have access to physiotherapy*. *Sometimes we can get access to psychology*. *Doctors can be a bit hit and miss if I’m completely honest*. *Yeah*, *so it does feel like it’s a very dietitian only approach*…*”* (PT 11)

*Healthcare systems provide barriers and enablers to nutritional management*. A number of barriers and enablers to providing best practice nutrition care were outlined by dietitians. Issues regarding food service, patient preferences and limited education and knowledge of dietary patterns were highlighted as potential barriers to implementing a dietary patterns approach into practice. Food service was highlighted as both a barrier and enabler in the inpatient setting:

“*In my work*, *food service issues are huge*. *Patients might be ordering things that you speak about*, *but they might not get them*. *Or they come and they’re cold*, *or they come in*, *they sit on a tray outside their room*, *because they’re in precautions*, *and no one takes it in*. *So even if we had the best guidelines for what best practice was*, *actually getting it to be implemented could be challenging*.*”* (PT 13)

Another dietitian highlighted the benefits of food service for patients who are not food secure:

“*We have some patients who eat everything*, *they love it*. *Being in the public system*, *some people don’t have that food security*. *It works well for them*…*”* (PT 14)

Financial barriers were also indicated by dietitians. In particular, in the private healthcare setting where patients pay for access to a dietitian:

“*They’ve already got so many costs when*, *you know*, *nothing’s free when they come in here*. *They are already paying the doctors a fortune and everything costs*. *And so yeah*, *they don’t always see the benefit of seeing a dietitian or they can’t justify the cost*, *or they just don’t want any more appointments*.*”* (PT 8)

Dietitians highlighted practical barriers and enablers to implementing a dietary patterns approach into their practice. In particular, dietitians discussed the need for greater professional education and evidence-based resources outlining potentially optimal dietary patterns and the importance of a strong evidence base to support their implementation into practice:

“*I think just the fact that we are required to be keeping up with evidence-based practice and you know*, *if there is strong evidence for [dietary patterns]*, *then that’s what ideally*, *we should be moving towards*. *And I think if it can get incorporated into guidelines or into or resources for dietitians*, *and for the general public*, *then I think that would be a great enabler for that to happen”* (PT 2)

However, dietitians suggested that the current nutrient-focussed approach to managing malnutrition (i.e., recommending foods based on their energy and protein content alone) was longstanding, which may hamper any suggested changes in practice:

“*I think one of the barriers would be that mindset of the dieticians and we’ve always done it this way*. *Particularly*, *your dietitians who have been working for 10 plus years*, *I think*, *trying to change those habits*.*”* (PT 11)

## Discussion

This qualitative study involving 14 oncology dietitians showed that the nutritional management of patients with malnutrition was consistent with nutrient-specific recommendations from evidence-based guidelines. A ‘food-first’ approach, or the enhancement of energy and protein intake primarily via the consumption of foods and beverages, was strongly valued by dietitians. However, dietitians’ food-based recommendations varied widely. Overall, there was limited awareness of dietary patterns among dietitians, with views varying as to whether they are relevant to the oncology setting. High-quality evidence for the role of dietary patterns in preventing and managing malnutrition and accompanying professional education were identified as enablers to implementing a dietary patterns approach into practice.

The findings of this study suggest that the dietary recommendations of oncology dietitians are congruent with evidence-based nutrition guidelines for people with cancer. In particular, oncology dietitians emphasised the importance of increasing energy and protein intake to maintain nutritional status and attenuate muscle loss [8,9]. The emphasis on energy and protein intake is consistent with the practice of dietitians who treat malnutrition in other high-nutritional risk cohorts (i.e., elderly adults) [30]. In a survey of 160 Australian dietitians working with elderly adults, 83% of dietitians focussed their management of malnutrition on improving both energy and protein intake using oral nutrition supplements, snacking and fortification of meals [30]. Current evidence-based nutrition guidelines for people with cancer recommend increased energy (25–30 kcal/kg/day) and protein intake (1–1.5 g/kg/day) to meet the increased requirements of people with cancer [9]. As such, consistency between dietitians current recommendations and dietary guidelines was evident.

We identified two distinct approaches used by oncology dietitians to manage malnutrition in practice. The approaches termed ‘any tolerated foods’, captured by the quote “any calorie is a good calorie”, or ‘foods beneficial for longer term health’ were both high-energy and high-protein recommendations, but varied significantly in regard to diet quality. One potential reason for this dichotomy of dietary approaches is a lack of evidence identifying the optimal dietary pattern(s) for people with cancer [22], and limited clear guidance as to which of these two approaches may be preferred during cancer treatment. As a result, oncology dietitians reported relying on long-standing beliefs around food intake, referring to knowledge from university (i.e., “…*long-standing knowledge from uni days…”)* to support food-specific recommendations. In contrast to the limited guidance for dietary patterns during the treatment period when risk of malnutrition is greatest, cancer survivorship guidelines provide information on specific foods and dietary patterns recommended to improve recurrence and survival after cancer diagnosis [31]. For example, the consumption of a healthy diet, rich in fruits and vegetables, legumes, and wholegrains, with limited consumption of red and processed meats, sugar-sweetened beverages, highly processed foods, and alcohol is in line with current cancer prevention recommendations [31–33]. As such, high-quality studies are required to determine the optimal dietary pattern(s) to achieve energy and protein requirements and manage malnutrition during cancer treatment, so that dietitians and their patients can make informed dietary decisions.

Several barriers to the implementation of dietary patterns into dietetic care emerged from the interviews with oncology dietitians. A key barrier was a perceived uncertainty regarding the relevance of dietary patterns to manage malnutrition in certain clinical situations. Practical factors such as the financial cost to patients for seeing a dietitian in the private healthcare sector, food-insecurity of patients when leaving a hospital setting, and psychosocial factors such as food preferences were highlighted by dietitians and have emerged as barriers to recommending dietary patterns in other chronic disease settings [34,35]. Consistent with barriers in other patient groups [35], oncology dietitians in this study discussed the importance of providing recommendations that are appropriate to the patient’s clinical situation and individual preferences. When barriers to oral intake, such as the presence of nutrition-related side effects, are high, dietitians questioned the relevance of a dietary pattern approach. As evidence for the role of dietary patterns in the clinical oncology setting develops, these findings emphasise the need to educate dietitians on the potential role of dietary patterns as one potential approach to managing malnutrition during cancer treatment and into survivorship. Continued professional development and educational resources are needed to provide guidance on how to adapt dietary patterns to meet individual and cultural preferences and clinical situations (e.g., food texturization). Such materials will aid in better integration of dietary pattern approaches into clinical practice, as seen in previous literature [34].

### Implications for future research and practice

There is currently insufficient evidence regarding the role of dietary patterns for cancer-related malnutrition to implement this approach into practice. Instead, future high-quality research should aim to identify and describe the optimal dietary pattern(s) to prevent and treat malnutrition across various cancer groups. The efficacy and tolerability of such dietary patterns should be examined during cancer treatment before incorporating this approach into dietary guidelines. Given the limited discussion of dietary patterns in the context of muscle loss in the present study, future research must consider potential effects of dietary patterns on muscle mass. Different cancer types may elicit unique nutrition-related side effects and require unique nutritional management strategies (e.g., swallowing difficulty for patients with head and neck cancer). Therefore, as the evidence for dietary patterns in managing malnutrition grows, more nuanced research (e.g., including specific cancer types) may be required to fully understand the current food-based recommendations, and barriers/enablers to implementing a dietary patterns approach of specialised (i.e., tumour specific) oncology dietitians. To support the implementation of a dietary patterns approach into practice, several required processes have been highlighted should there be sufficient evidence for a dietary patterns approach in future. Firstly, dietary patterns would need to be integrated into evidence-based dietary guidelines which are largely referred to by dietitians to inform their practice. Additionally, professional development and educational resources would be required to support dietitians’ understanding of dietary patterns and how certain dietary patterns may be tailored and beneficial to optimise their patient’s dietary intake when oral nutritional intake is possible. Whilst dietitians stated that dietary patterns were “too prescriptive”, dietary patterns would provide a framework for specific food-based recommendations that should be adapted to the patient’s clinical situation and food preferences. A similar approach has been adopted in cancer survivorship guidelines which consider the beneficial role of certain foods and dietary patterns for long term health [31,33].

### Strengths and limitations

Dietitians were interviewed from a range of clinical settings including public and private Australian healthcare centres. A robust data analysis method was utilised, three interview transcripts were open coded by three authors (ARC, NK and AU) in collaboration and subsequent transcripts were coded by a single author (ARC). Having one researcher code all transcripts enhanced consistently, whilst collaborative coding was beneficial in the interpreting complex data and reducing the likelihood that data were missed or misinterpreted. Data saturation was achieved, indicating that the sample of oncology dietitians was appropriate to address the aims of this study. However, the majority of dietitians were from tertiary healthcare centres (i.e., hospitals), limiting our understanding of dietetic practice and the resources needed to implement dietary patterns into the community or private healthcare settings. Dietitians who provided care to people with a broad range of cancer types were included in this study. Cancer type can strongly influence the optimal nutrition management strategy for a patient. For example, patients with head and neck cancer (to whom 29% of dietitians provided care) may be more likely to receive a prophylactic feeding tube as a primary source of nutrition due to the expected experience of nutrition-limiting side-effects. As such, some dietitians may have perceived dietary patterns as less relevant for the treatment of malnutrition. A multifaceted approach was used to recruit dietitians from multiple Australian states and territories and including those with varying levels of professional experience, this allowed for recruitment of a heterogeneous sample who could provide a broad understanding to this research. However, the recruitment strategies used, particularly recruiting dietitians through the professional networks of authors, may inadvertently introduce bias to the sample.

## Conclusion

The findings from this study indicate that oncology dietitians’ practice is in line with nutrient-focussed evidence-based nutrition guidelines. While all dietitians agreed that increased energy and protein intake is required to prevent and treat cancer-related malnutrition, there was ambiguity regarding the optimal food-based strategies to achieve these requirements in practice. In line with a paucity of evidence for dietary pattens and cancer-related malnutrition, dietitians lacked understanding of dietary patterns and perceived them as lacking relevance in the context of the acute treatment period. This study highlighted the need to overcome potential barriers in the healthcare system, such as adequacy of the food service provision, and a requirement for professional education to expand dietitians knowledge of dietary patterns and their potential use in the clinical oncology setting as literature on this topic expands. Further research into the role of dietary patterns to prevent and treat cancer-related malnutrition is needed and may help to inform beneficial food-based strategies to best patient care.

## Supporting information

S1 Table COREQ checklist. COREQ Checklist with reference to in text information.
(PDF)
